# Effect of body orientation and joint movement on local bioimpedance measurements

**DOI:** 10.2478/joeb-2024-0016

**Published:** 2024-10-05

**Authors:** Sisay Mebre Abie, Alejandro Ortega de Román, Jie Hou

**Affiliations:** 1 Department of Physics, University of Oslo, 0316 Oslo, Norway; 2 Department of Earth Sciences and Condensed Matter Physics, University of Cantabria 39005, Santander, Spain

**Keywords:** Bioimpedance, Ultrasound, Skin layer characterization, Body position

## Abstract

The purpose of this pilot study was to determine if body orientation, skin treatment, joint angle, and shoulder arch movements affect localized bioimpedance spectroscopy (BIS) measurement. Nowadays, there are various wearable and portable impedance measurement tools in different shapes and sizes. Therefore, the body position and orientation of the subject during measurement may be of great importance for the comparability of the results. Ultrasound machine was used to measure the thickness of the skin layers and then bioimpedance measurements were performed for ten young men (age=23 ± 5) at room temperature (22°C) for different body orientations, skin treatments, joint angle, and shoulder arch movements. The results were analyzed using statistical methods and graphical presentation using Python and MatLab. Our observations indicate that there is a significant difference between standing straight up, supine and sitting positions. The results show that there is a significant difference between the two skin treatments (alcohol vs tape stripping). Moreover, joint angle and shoulder arch movements also have an impact on the impedance data. Therefore, to be able to control these factors can potentially improve the quality and comparability of the measured impedance data.

## Introduction

Bioimpedance analysis (BIA) is a widely used noninvasive, low-cost, and reliable method that has firmly established itself as an appropriate modality to evaluate body composition and body fluid distributions [1, 2]. The evaluation of body water provides practical and important information to the health fitness professional, diagnostics and prognostics in clinical care [3, 4]. Possible uses for a sensor that measures changes in fluid volume include a variety of sports, different patient populations (kidney failure, heart failure etc.), the military, and a number of physically demanding professions [5]. It measures the electrical properties of biological tissue and utilizes different tissue types that have different electrical properties due to their structure and composition.

Electrical bioimpedance (Z, Ω) is defined as the opposition of a conductor to the flow of an alternating electrical current applied to it. Bioimpedance is a complex parameter derived from the vector relationship between resistance (R, Ω) and reactance (Xc, Ω). Therefore, the physical basis of the bioimpedance method is that the biological tissue is a network of resistors and capacitors with the fluids behaving as resistors and cell membranes acting as capacitors. Thus, the body may be represented as a parallel resistor-capacitor (RC) equivalent circuit in which the introduced alternating current divides into resistive (fluid and electrolytes) and capacitive (cell membranes and tissue interfaces) pathways [1, 6].

Nowadays, Bioimpedance is applicable in different sectors including medical science, such as determining body composition. However, in recent decades it has gained more and more attention in the measurement of fluid balance [7]. Moreover, bioimpedance technology offers valuable tissue/cell physiology and pathology information. Bioimpedance sensor technology is becoming the basis of novel and noninvasive medical diagnostic devices [8]. Despite several applications of bioimpedance technology in different fields of study, it has a limitation on its application in clinical practice and significant research is required to address the challenges.

Recent advancements in the field of microelectronics have led to the miniaturization of bioimpedance devices which also has enabled the development of wearable devices that can record bioimpedance signals [9, 10]. Furthermore, different shapes and sizes of sensors are developed that allow continuous use. However, due to its modest size, these sensors can only measure a small part of the body. It is therefore crucial to map how local bioimpedance measurements are affected by various factors that may have a lesser effect on the measurements of whole-body devices. This includes factors such as altered perfusion in the measurement area (for example due to temperature change), individual variation in tissue composition at the measurement site, change in body orientation, muscle contraction in the measurement area and changed angle in nearby joints [11, 12, 13].

The main objective of this study is to investigate the impact of the following factors on local bioimpedance measurements:

- Underlying tissue composition (skin layer thickness).
- Body orientation.
- Changed joint angle.
- Different skin preparation procedures.

The above-mentioned investigation was made by assuming that other factors that may affect the impedance measurement are constant. To minimize the effect of age difference, the subject’s age was limited to healthy adults between 20 to 30. To minimize the effect of temperature, the subjects were asked to rest at room temperature for a few minutes to ensure that the test subjects were in a relaxed and calm state before the onset of the experiment.

## Materials and methods

### Participants and measurement setup

A total of 10 young healthy males participated in this study. After obtaining approval for the study from the local ethics committee, all subjects participated in the study after reading and signing the consent form. The subjects were asked to rest for a few minutes before the measurement started and underlying tissue (skin) layer thickness measurement was taken using ultrasound (General Electric (GE) Vivid E9 Ultrasound system) at the measurement site (without any skin treatment other than ultrasound gel).

Then bioimpedance was measured on the right shoulder using the Zurich Instruments MFIA impedance analyzer (Zurich Instruments AG, Switzerland). Four electrode configuration with Skintact ECG electrodes (MedEnvoy, Switzerland) were used and a sweep between 10 Hz and 1 MHz consisting of 40 frequencies was performed. As shown in the picture 1, the electrodes were attached on the right side of the shoulder. The participants were asked to complete a series of tasks over one-hour period.

### Experimental protocol

Each subject underwent several bioimpedance measurements with different body orientations and positions of joint angle. After each measurement, the subject was instructed to change the body orientation and position of the arm and stay in the position for at least four minutes to give the new condition time to stabilize.

**Figure 1:**
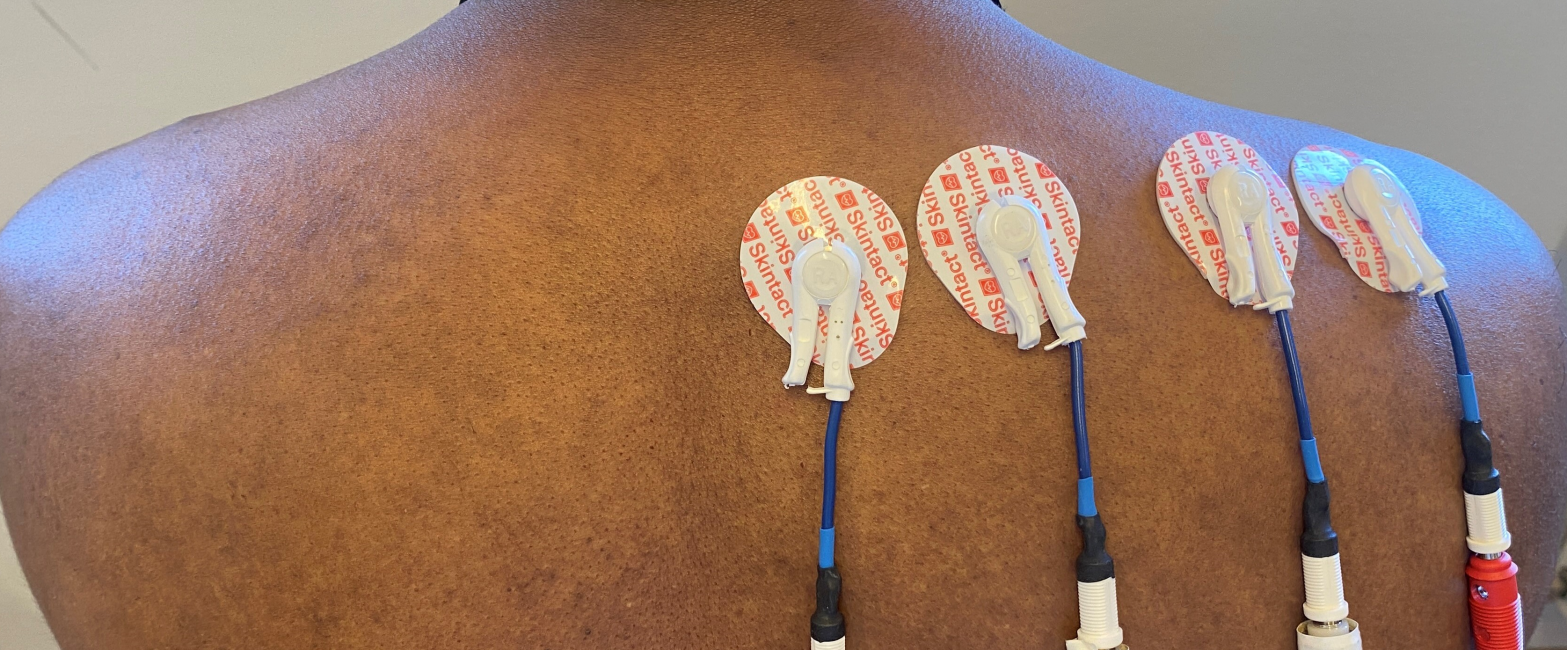
The electrode placement during the measurements.

Bioimpedance measurements are dependent on the posture and the level of movement of the subjects [13]. Different research groups reported that body orientation has a great effect on bioimpedance measurements, both segmental and whole-body measurements [11, 12]. Here we evaluate the precision and accuracy of bioimpedance measurements in the standing position, compared to the supine position which is commonly used in clinical practice. First, the measurement was made in a standing position and then in a supine position. The latter was carried out after the subject had been lying down for approximately 4 minutes. The whole procedure lasted about 8 minutes. The lying position has to avoid creating any pressure on the attached electrode (lying on the electrode-free side of the body).

To explore the effect of a modified joint angle, the shoulder joint and shoulder arch movements were assessed while sitting. Throughout all the measurements, the electrode remains attached. There is an assumption that bioimpedance and muscle condition changes can occur if the angle in a nearby joint moves. For example, moving the joint angle can change the thickness of the muscle layers under the electrode, and also create movement of the skin in relation to the underlying tissue. For the back position, both movements in the shoulder joint and the shoulder arch could affect the tissue composition in the measurement area. During the measurements, each position was held for 4 minutes. For all positions, to minimize the effect of muscle contraction, the arms were held in position with the help of study personnel/equipment, not with the subject’s own muscle power.

Furthermore, to investigate the effect of different skin preparations on bioimpedance measurement, we treated the skin with tape and alcohol right before the attachment of the electrodes.

**Table 1:** Protocol of body orientation, shoulder joint and shoulder arch movement for bioimpedance measurement.

Number	Body orientation, shoulder joint and shoulder arch movement
**1**	Standing straight up position
**2**	Supine position
**3**	Sitting and arms straight down and relaxed - starting position (arms straight down)
**4**	Arms straight forward (90° shoulder flexion)
**5**	Starting position (arms straight down)
**6**	Arms behind the back (slight extension in shoulder joints)
**7**	Starting position (arms straight down)
**8**	Arms straight out to the side (90° abduction in the shoulder joint)
**9**	Starting position (arms straight down)
**10**	Arms straight back (full extension in shoulder joint, retraction in shoulder arch)
**11**	Starting position (arms straight down)
**12**	Arms straight up (180° shoulder flexion)
**13**	Starting position (arms straight down)

### Informed consent

Informed consent has been obtained from all individuals included in this study.

### Ethical approval

The research related to human use has been complied with all relevant national regulations, institutional policies and in accordance with the tenets of the Helsinki Declaration, and has been approved by the authors’ institutional review board or equivalent committee.

## Results

**Figure 2:**
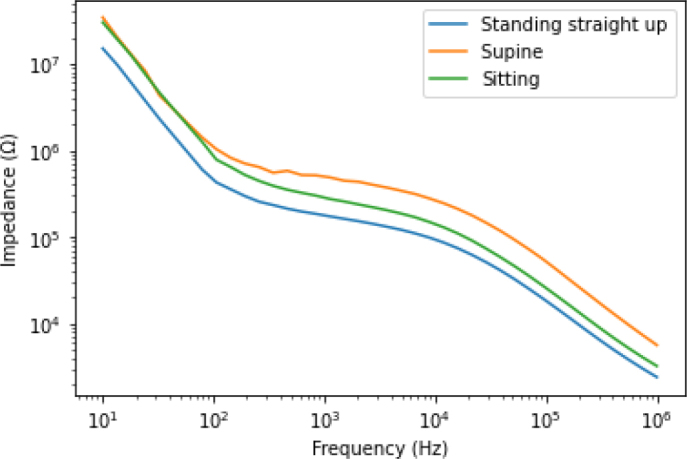
The mean of the bioimpedance measurement results of all volunteers for the first three body postures, i.e., standing straight up, lying down in supine position, and sitting with arm down and relaxed.

The subject’s position during the test may be of significant importance for the comparability of the results. Here we evaluate the effect of bioimpedance measurements in the standing and sitting position, compared to the supine position which is commonly used in clinical practice. Figure 2 shows the impedance measurement result of the three different body orientations (standing straight up, sitting, and supine). As shown in Figure 2, there were detectable differences between the measured bioimpedance of the three body orientations (supine, standing straight up, and sitting) except for the supine and sitting in the lower frequency range. The value of the impedance measurement was the highest in the supine position, while the lowest was in the standing position.

**Figure 3:**
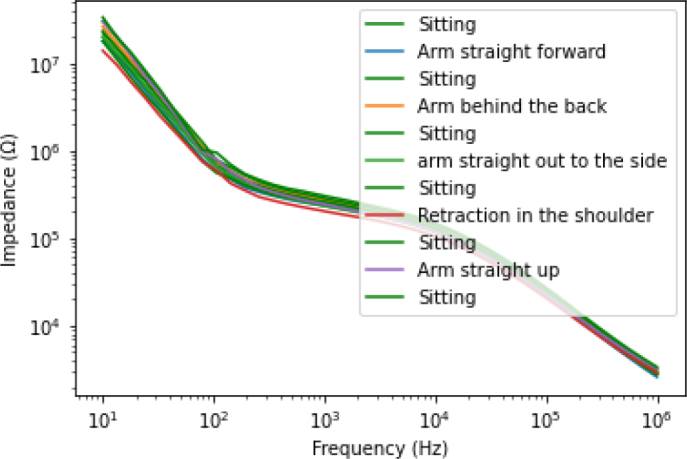
The mean of the bioimpedance measurement results of all subjects for each body posture. The 6 green legends “Sitting” refers to the measurement made after each movement (shoulder joint and shoulder arch movements), the subjects were asked to return to the starting position (Sitting and arms straight down and relaxed).

Furthermore, we also assessed the effect of different joint angles and shoulder arch movements on the bioimpedance results. Figure 3 presents the results during different movements of the joint angle and different shoulder arch. As Figure 3 shows, the joint angle and the shoulder arch movement also have a certain effect on the impedance measurement results. However, the impedance of the sitting position measured after the different joint angles and shoulder arch movements overlapped. This indicates that for a calmly seated person, the value of the impedance remains fairly the same. There were no significant variations among these measurements. Henceforth, we only use one of the sitting measurement data in further figures.

One of the key challenges for bioimpedance-based measurement systems is to understand the effect of the composition of the tissue under test. Before attaching the electrodes on the right shoulder, skin tissue layer thickness was assessed with ultrasound. Due to the limited resolution of the scanned ultrasound image, the epidermis and dermis were together labeled as layer 1, and the hypodermis and the subcutaneous fat were labeled as layer 2. Table 2 shows the measured thickness of the skin layers of all ten participants.

**Figure 4:**
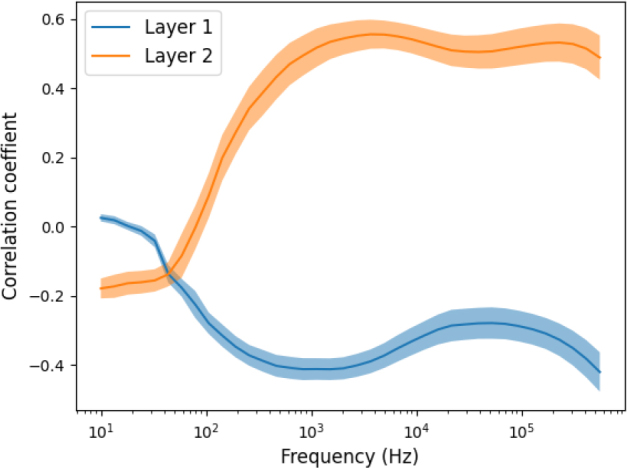
The correlation coefficient between the impedance measurement result and the two skin layer thickness. Pearson correlation was performed between 8 different joint angles and shoulder arch positions and the two skin layer thicknesses.

**Figure 5:**
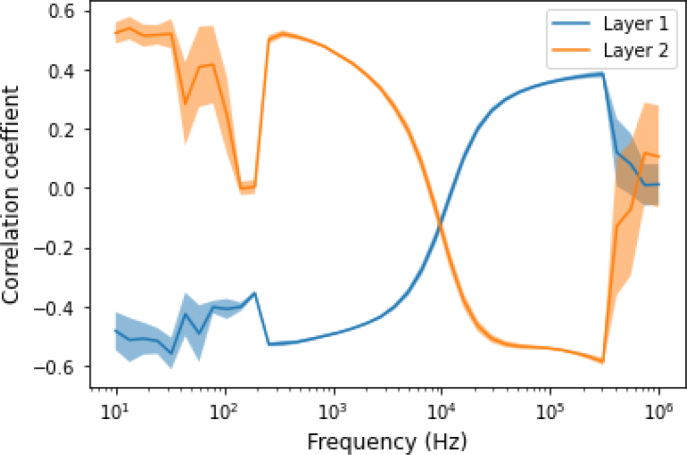
The correlation coefficient between the phase angle measurement result and the two skin layer thickness. Pearson correlation was performed between 8 different joint angles and shoulder arch positions and the two skin layer thicknesses.

A statistical Pearson correlation analysis was performed to assess the effect of tissue composition (tissue layer thickness) on bioimpedance measurement. Figure 4 and 5 show the correlation coefficient of the two skin layers with the value of the impedance measured in different body orientations and different joint angles and shoulder arch movements.

**Table 2:** Thickness of the two distinguished layers of skin on the right shoulder tissue which were examined using the ultrasound machine for the 10 volunteers. Layer 1 is epidermis and dermis and layer 2 is hypodermics/subcutaneous tissue.

Participant	1	2	3	4	5	6	7	8	9	10
**Layer 1 (cm)**	0.20	0.25	0.25	0.15	0.15	0.15	0.20	0.10	0.20	0.25
**Layer 2 (cm)**	0.15	0.27	0.15	0.50	0.35	0.70	0.75	0.40	0.15	0.25

The thickness of the inner layer (layer 2) had a moderate positive correlation (r = 0.5 ± 0.1) to the impedance measurement, especially in the higher frequency range. However, it had a weak negative correlation at lower frequencies and became zero around 100 Hz. Then, this correlation increased up to 1 kHz and became moderate positive correlation. Similarly, the outer skin layer (layer 1) has a moderate negative correlation (r = -0.5 ± -0.1) with the impedance value. However, there was no correlation (r = 0) in the lower frequency up to 100 Hz. This correlation increased starting from 100 Hz until 1 kHz and then it became moderate negative correlation in the higher frequencies.

Furthermore, a correlation analysis was performed between the phase angle and the two skin layer thickness. Figure 5 shows the correlation coefficient between the two skin layer thicknesses and the phase angle for the whole frequency range. As the figure indicates, the correlation depends on the frequency, at lower frequencies, there was a moderate negative and positive correlation (r = 0.5 ± 0.1) between the phase angle and the thickness of skin layer 1 and layer 2 respectively. At higher frequencies, there was a positive and negative moderate correlation (r = 0.5 ± 0.1) between the phase angle and the thickness of skin layer 1 and layer 2 respectively. However, this correlation disappeared around 10 KHz.

**Figure 6:**
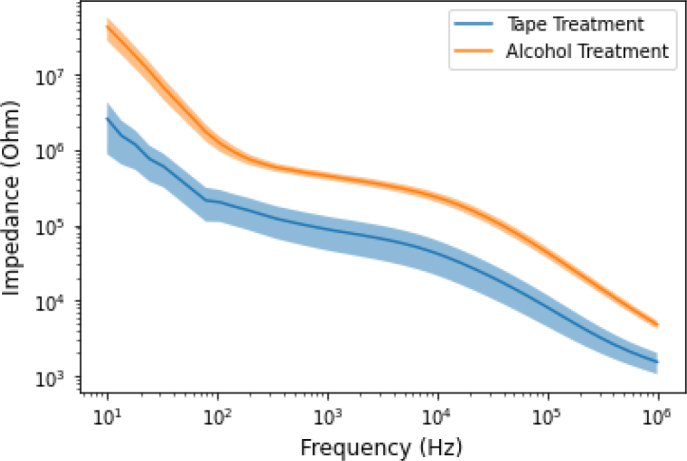
The comparison between the impedance measurements of the two skin treatments. The shaded area is the 95% confidence interval, the solid line in the middle represents the mean value of each group.

**Figure 7:**
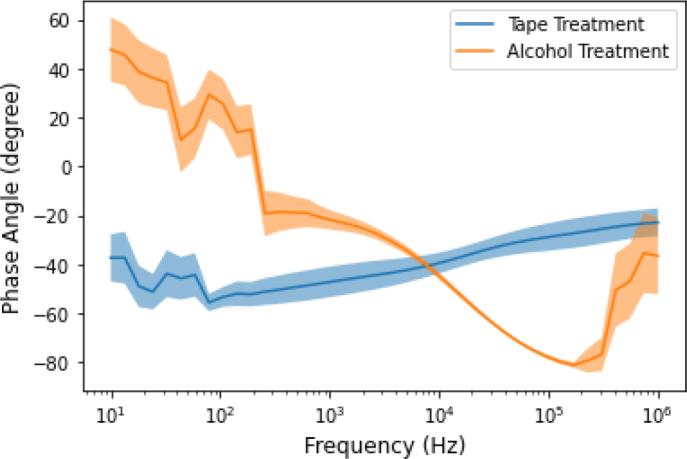
The comparison between the phase angle measurements of the two skin treatments. The shaded area is the 95% confidence interval, the solid line in the middle represents the mean value of each group.

We tested the effect of different skin treatments on the bioimpedance measurement. The subject’s skin was treated by two different methods (Tape and Alcohol) before attaching the electrode for measurement. The comparison of alcohol and tape-treated skin bioimpedance measurement and corresponding phase angle results for each frequency and each body posture was made using graphical observation and statistical analysis. Figure 6 and 7 show the 95% confidence interval which includes the mean value of each group. There was a detectable difference between the two treatments in both impedance modules and phase angle. The measurement value of the impedance in the alcohol-treated skin was higher than the tape-treated impedance measurement value.

**Figure 8:**
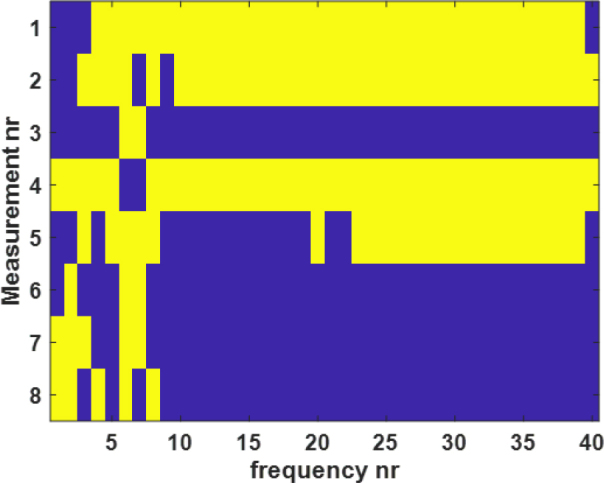
The comparison between the impedance measurements of the two skin treatments. The Wilcoxon rank sum test was performed between 8 different joint angles and shoulder arch positions of the two skin treatments before the attachment of the electrode.

Moreover, non-parametric t-test (Wilcoxon rank sum test) was performed with MATLAB (version R2020a, The MathWorks Inc., Massachusetts, USA, 2020) between the alcohol and tape-treated skin impedance measured for the whole frequency range (from 10 Hz to 1 MHz with 40 frequency points). The result is shown in Figure 8, the yellow color (logical value h = 1) indicates a rejection of the null hypothesis of equal medians, and the blue color (logical value h = 0) indicates a failure to reject the null hypothesis at the 5 percent significance level. Because the sample sizes are small (five each), rank sum calculates the p-value using the exact method (The Wilcoxon-test function will calculate exact p-values). Otherwise, a normal approximation is used. Rank sum computes the two-sided p-value by doubling the most significant one-sided value.

Based on the p-value obtained and the logical values, there was enough evidence to reject the null hypothesis. The results showed a significant difference between the median of the tape- and alcohol-treated skin impedance measurement results. There was a significant difference between the two groups in the lower and higher frequencies and no significant difference in the middle frequencies.

Lastly, the Wilcoxon rank sum test was performed on the phase angle measured between the treatment with alcohol and tape. There was a significant difference between the two groups at nearly every frequency point during standing, supine, and sitting position. Whereas for the measurement of joint angle and shoulder arch movement, there were significant differences between the two groups in the lower frequencies. There were no significant differences between the phase angle for the two different skin treatments for frequencies above 10 KHz.

## Discussion

This study analyzed the relationship between the skin layer thickness, body position, shoulder joint and shoulder arch movement and bioimpedance measurement data. The human body is mainly composed of water, proteins, fat, glycogen, and minerals. Changes in body tissue structure and composition occur during growth, maturation, aging, diseases, and other activities. Therefore, there is considerable inter- and intra-individual variability. In general, bioimpedance varies with tissue composition and structure as well as with the frequency of the applied current [14]. Before the electrodes were attached for impedance measurement, ultrasound was used to do skin layer characterization at the measurement locations, which involved measuring the thickness of skin including subcutaneous fat. The ultrasound measurements were performed before the skin treatments.

The skin layer thickness is dependent on the location of the scan. Because there are scapula bones in the shoulder position, the skin layer was characterised in an area where the bone did not exist. As shown in table 2, the thickness of both layers varies from subject to subject. The average thickness of skin layers 1 and 2 in this study is 0.19 ± 0.05 cm and 0.367 ± 0.22 cm (N = 10) respectively.

According to earlier studies, the thickness of the skin depends on the body site. For instance, the epidermal skin thickness is thickest on the palms of the hands and soles of the feet, and thinnest in the face (eyelids) and genitalia [15, 16, 17]. The variation in epidermal skin thickness is mostly due to the variation in the stratum corneum thickness, where it is approximately 10-20 μm thick in general, and it can be as thick as 2 mm on the palms of the hands and feet [18, 19]. Similarly, the thickness of the hypodermis varies in different regions of the body and in different people. In men, the hypodermis is thickest in the abdomen and shoulders. In women, it is thickest in the hips, thighs, and buttocks [20, 21].

Using the measured skin layer thickness and the impedance data, a correlation assessment was performed, we found different degrees of correlations (from very low to moderate) between the two skin layers and the impedance data. As shown in Figure 4, at lower frequencies (<1 KHz), the correlation was minimum, as the frequency increased the correlation started to increase to moderate (r=0.6) and remained high for the rest of the frequency range.

Correlations of the skin layer thickness with the bioimpedance data in our study ranged from moderate, from about r = ± 0.6, to almost r = ± 0.2. Other studies have also reported correlations on different skin layers with bioimpedance measurements [11]. The negative correlation between skin layer 1 (outer skin) and impedance is likely due to the very dense nature of the outer skin layer, which acts as a barrier against radiation, chemicals and pathogens whilst limiting water loss through the skin. The impedance is also dependent on the moisture and the temperature of the skin. The positive correlation of skin layer 2 (inner skin) with the impedance is likely due to the high content of fat (which acts as an insulator) and loose connective tissue. These results also match with the previous reports [15, 22, 23]. According to Sung et al. [22], there were significant positive correlations between the subcutaneous fat layer thickness and the resistance and phase, whereas the reactance was minimally affected by the subcutaneous fat.

Similarly, the correlation of the two measured skin layer thickness and the phase angle of the impedance measurement varied from no correlation to moderate correlation (r = ± 0.6). As shown in Figure 5, the correlation coefficient depends on the frequency. There was no correlation in the middle frequency (10 KHz) and frequencies above 300 KHz, whereas there were positive and negative correlations at the lower (less than 1 kHz) and higher (between 50 KHz and 300 KHz) frequencies. This indicates that different frequencies have different interactions with the different cellular components that are found in the two skin layers. It is known that changes in impedance arise from changes in the nature of the conducting medium (such as the skin, subcutaneous fat layer, muscle, and other connective tissues). However, it is unclear how the different frequencies of the applied signal interact and are affected by the different components of the tissues.

Furthermore, there are several factors that can affect the bioimpedance analysis results, such as non-standardization of body position and orientation, previous physical exercise, and food or fluid intake [15, 14, 17]. The position of the patient’s body and the movement of the joints during the test are of great importance.

Physiological changes occur during physical exercise that can influence impedance measurements and distort results. Some researchers have emphasized the advantage of lying over standing due to the better equalization of fluid levels [24, 25, 26]. Therefore, one of the objectives of the current study was to assess the impact of the body position and the movement of the joint angle on the localized muscle, as well as to observe the changes in the electrical impedance data under different frequencies. Thus, we compared the effects of different body positions and joint angle movements on the bioimpedance measurement output and found that there are detectable differences between the three body positions. Comparisons of impedance data from different body orientations reveal differences in tissue electrical properties, which is consistent with previous findings [11]. These differences in electrical properties might be due to the fact that different joint angle movements have different effects on surrounding muscle thickness and fluid distribution. Each movement at a joint results from the contraction or relaxation of the muscles attached to the bones on either side of the articulation.

**Figure 9:**
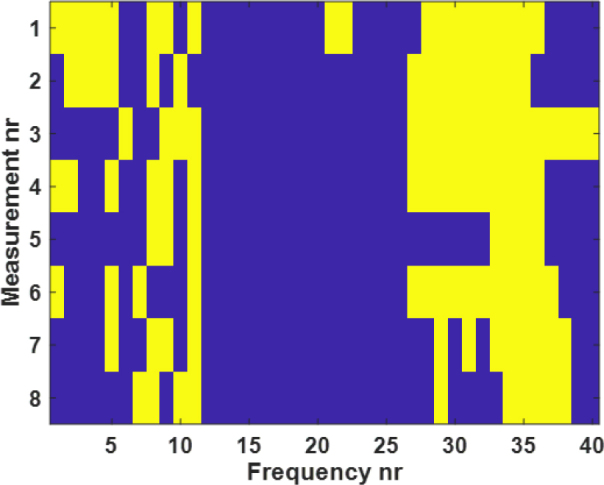
The comparison between the phase angle measurement of the two skin treatments. The Wilcoxon rank sum test was performed between 8 different body positions, joint angles, and shoulder arch positions of the two skin treatments before the attachment of the electrode. In the y-axis, the number represents: 1-Standing straight up; 2-supine; 3-sitting; 4-Arms straight forward; 5-Arms behind the back; 6-Arms straight out to the side; 7-Arms straight back; 8-Arms straight up.

The other factor that has not received that much attention so far is skin treatment. The subject’s skin was treated before the electrodes were attached for impedance measurement. In this study, alcohol and tape-stripping were used to clean the skin. For each frequency, a non-parametric t-test (Wilcoxon rank-sum test or Wilcoxon–Mann–Whitney test) was performed to compare the impedance and phase angle of the two treatments. As shown in the figure 8 and 9, significant differences were found between the impedance and phase angle for the two treatments. These differences were observed in nearly every frequency during standing, supine, and sitting position. For the measurements of the joint angle and shoulder arch movement, significant differences were observed in the lower frequencies (< 10 KHz). However, noticeable differences were found only in the lower (< 10 KHz) and higher (> 100 KHz) frequencies of the phase angle of the two skin treatments. As Figure 6 and 7 showed, there were noticeable differences in both impedance and phase angle data between the two skin treatments. A higher impedance was obtained by alcohol treatment and a lower impedance by the tape treatment. The reason for this phenomenon can be due to the fact that tape stripping removes some of the dead cells of the skin layer which contributes to the increment of the impedance values. Removing a small quantity of dead skin cells had a big impact as shown in Figure 6. Based on these results and an earlier study by Lawler et.al. [27], we conclude that the removal of the stratum corneum has profound effects on the impedance and phase angle. Alcohol treatment removed saline ions which are highly conductive and facilitate the flow of electric current, resulting in an increase in impedance. In addition, ethanol is a poor conductor of electricity compared to saline solution. When the saline solution is replaced by alcohol, the ionic pathway that allows current to flow gets disrupted, leading to a higher impedance. In summary, the detected skin impedance is closely related to the thickness of the outermost skin layer epidermis (stratum corneum). Also, the impedance of the stratum corneum occupies a huge proportion of the total skin impedance. Thus, the treatment of stratum corneum greatly affects the measured skin impedance.

## Conclusion

This pilot study highlighted the relevance of studying the impact of body orientation, skin treatment, joint angle and shoulder arch movements on bioimpedance measurements. We showed that body orientation, joint angle movement, and skin treatment can have effects on the impedance measurement results. These effects also depend on the frequency applied. The results verify the sensitivity of bioimpedance measurements to changes in the underlying skin properties and, therefore, to body orientation during impedance measurement. As a pilot study, further investigation is needed to understand the full scope of how these factors may influence bioimpedance measurements.
